# Home-based care for people living with dementia at the end of life: the perspective of experts

**DOI:** 10.1186/s12904-023-01251-z

**Published:** 2023-09-01

**Authors:** Christiane Pinkert, Bernhard Holle

**Affiliations:** 1 Deutsches Zentrum für Neurodegenerative Erkrankungen, Standort Witten, Witten, Germany; 2 Faculty of Health, School of Nursing Science, Witten/Herdecke University (UW/H), Witten, Germany

**Keywords:** Dementia, Palliative care, Community care, Expert interview

## Abstract

**Background:**

In the last phase of their lives, people living with dementia often indicate restlessness, anxiety or pain. Further, their care is considered inadequate, as they are, for example, sometimes overtreated for curative care or undertreated for pain management. These patients also face multiple barriers in accessing palliative care. This qualitative study explores the perception of experts about how people living with dementia in Germany are cared for at home toward the end of their lives.

**Methods:**

A total of 12 experts involved in outpatient/palliative care were recruited to constitute a purposive, heterogeneous sample. Interviews, which were structured using an interview guide, were conducted with physicians, nurses, representatives of health insurance funds, welfare associations, municipal counselling centres, scientists and coordinators of outpatient palliative care and voluntary work; the interviews were transcribed and analysed via thematic content analysis, based on Kuckartz’s method.

**Results:**

The analysis of the results led to the establishment of four main categories that focused on *formal care arrangements, the roles of relatives in care arrangements, the specifics of dementia, and restrictions on access to palliative care.*

**Conclusions:**

Suitable end-of-life care for people living with dementia and support for their relatives require resources and the conceptualisation of specific care arrangements to help minimise potential barriers that prevent access to palliative care.

**Supplementary Information:**

The online version contains supplementary material available at 10.1186/s12904-023-01251-z.

## Background

Approximately 55 million people worldwide currently live with dementia [1]. Dementia is an incurable, life-limiting and progressive disease. It is the seventh leading cause of death globally [2] and the second leading cause of death in high-income countries [3]. While people living with dementia in the early and middle stages of the disease are often primarily affected by a decline in cognitive and physical abilities, loss of autonomy and personality changes, additional symptoms may occur in the last phase of their lives. These symptoms include restlessness, anxiety, pain, shortness of breath and difficulty swallowing [4–7]. Addressing these distressing conditions, maintaining or enhancing the well-being of this population and dealing with psychological, spiritual and social needs are all part of palliative care [8].

Experts consider that people living with dementia need specific palliative care, as the course of the disease and the limitations they face, especially in terms of decision-making ability and communication, differ significantly from the limitations of people with other life-limiting diseases, such as cancer. Furthermore, coping with behaviour that challenges is a major issue for both family and professional carers [9]. Therefore, the European Association for Palliative Care has supplemented the general principles applied to palliative care for people with a terminal illness to optimize palliative care for people living with dementia based on evidence and expert consensus [9]. Eleven domains were developed, which include a total of 57 recommendations on topics such as “education of the health care team” or “avoiding overly aggressive, burdensome or futile treatment”, among others. However, a review by Miranda et al. [10] showed that these criteria are rarely considered in the palliative care of people living with dementia who reside at home. Overall, the treatment and care of people living with dementia at the end of life is considered to be inadequate [11–13]. On the one hand, people living with dementia are “at risk of overtreatment with burdensome and possibly non-beneficial interventions” [14], such as artificial feeding, restraints, laboratory tests and the administration of intravenous drugs. On the other hand, these patients receive too few necessary palliative interventions, such as adequate pain management, emotional and social support, spiritual care and support for their family carers [13].

Worldwide, the majority (84%) of people living with dementia are cared for by informal and/or formal carers at home [15]. In Germany, approximately 70% of people living with dementia over the age of 80 are cared for at home [16]. Especially in the care of people living with dementia at home, relatives play an important role. They are the ones who take over some or all of the care, who organise professional care and who have to make decisions on behalf of their family member. In this context, family carers are emburdened with difficult access to or lack of support services, difficulties in navigating the health care system, as well as a lack of communication between family members themselves and care providers. Additionally, they find it a burden to struggle with health insurance and care providers in order to find appropriate care and support [17, 18].

International studies on places of death indicate that people living with dementia are often not cared for at home at the end of life or do not die at home [19–21]. However, there is great international variability in this regard; in a study of 14 European and non-European countries, the proportion of people with dementia dying at home ranged from 3.8% in the Netherlands to 69.3% in Mexico [21]. There are also inconsistent results for Germany. The numbers range from 19.9% [22] to 36.2% [23] to 42.4% [6] for the percentage of people living with dementia who die at home. According to these studies, most people living with dementia in Germany die in nursing homes, followed by hospitals [6, 21, 22], and very rarely in a palliative care unit or in a hospice [6, 22, 23].

In Germany, outpatient and inpatient palliative care is secured by legal regulations and anchored in the general system of health insurance [24]. The provision of palliative care has been greatly expanded in recent years, with a special focus on outpatient palliative care [25]. In Germany, palliative care is available at two levels: general and specialised palliative care. General palliative care is provided by the general practitioner and ambulatory nursing services and includes basic measures for the control of symptoms, collaboration with other service providers and communication with relatives. Specialised palliative care is dedicated to people with complex problems and needs because they require a wide range of therapeutic interventions to control their symptoms and address their psychological, social and spiritual problems; these services can only be provided by a multiprofessional team with appropriate qualifications [26]. It is unclear whether the structures and the qualifications of care providers are also tailored to the specific needs of people living with dementia. In the national dementia strategy for Germany, the stakeholders involved have committed themselves, among other things, to supporting relatives of people living with dementia in end-of-life care and to improving access to palliative care [27]. However, there have been few studies on palliative care for people living with dementia in Germany [7, 28–30], and little is known about how this population is cared for at the end of life, particularly in the outpatient setting.

### Objectives

To better understand how people with dementia living at home are cared for at the end of life the objectives of this study were to gain insight into (a) the organisation of end-of-life care for people with dementia living at home; (b) the role that family members play in end-of-life care; (c) the way people living with dementia access palliative care; and (c) the factors that enable or hinder a person living with dementia from remaining at home until the end of his or her life.

## Method

A qualitative design was chosen for the study to describe how people living with dementia are cared for at home at the end of their lives from the perspective of experts involved in outpatient palliative care and/or in caring for people living with dementia. Since little knowledge is available on this topic in the German setting it seemed appropriate to use a relatively open approach to interview experts in a structured way on the one hand and to give them the opportunity to set their own thematic priorities on the other.

### Participants and recruitment

For this study, a purposive sampling method was applied to obtain a broad overview of the care situation. Experts were defined as actors who were either involved in the direct care of people living with dementia at the end of life or who were concerned with the topic from the perspective of research, counselling or financing. Therefore, (palliative) physicians, nurses, representatives of outpatient hospice work, representatives of welfare organisations and health insurance companies, scientists and consultants from all over Germany were approached. Contacts with potential participants were mainly established through the researcher’s professional network. Selected participants were contacted by email or phone. More detailed information about the project was then often provided in a telephone conversation. The recruitment process proved to be difficult and lengthy (eight months) because almost all of the potential participants worked in direct patient care and were heavily involved in the COVID-19 pandemic at the time of recruitment.

### Data collection

An interview guide based on the research literature was used during the semi structured interviews (see supplementary file 1). The interview guide pertains to access to palliative care, challenges in the last phase of life, support for relatives, and factors enabling people living with dementia to remain at home. Demographic data about the interview participants were collected via a short questionnaire. After contacting and informing the participants, an appointment for the interview was arranged. Four of the participants and the interviewer already knew each other from professional contexts before the interviews. With the exception of two interviews, all other interviews were conducted via telephone due to contact restrictions during the COVID-19 pandemic. One interview took place as a face-to-face interview at the participant´s workplace, and one interview was conducted virtually. One interview involved two participants simultaneously, and all other interviews included only one person each. Interviews lasted between 16 and 60 min (median 55 min). All interviews were conducted in German and were audio-digitally recorded and transcribed verbatim. The interviews were conducted by the first author (CP) (a female registered nurse and postdoctoral researcher with many years of experience in qualitative research) between February and September 2020.

### Data analysis

The data were analysed by the first author using thematic content analysis according to Kuckartz [31]. This method allows for the development of both deductive and inductive categories. The analysis was divided into seven steps: 1. initiating text work, 2. developing the main categories, 3. coding the material, 4. collating all text passages coded with the same main category, 5. inductively developing subcategories, 6. recoding the material with the elaborate category system, and 7. carrying out complex analyses and visualisations. In the present study, the main categories were mainly derived deductively from the interview questions. The subcategories were developed inductively from the material. Owing to the relatively small amount of data, the last step of the analysis was omitted. Likewise, data collection and data analysis partly took place in parallel due to the lengthy recruitment process. However, the interview guide was not changed nor adapted on the basis of preliminary results. The software MAXQDA (version 2020, Verbi) was used to support the data analysis and to organise the data. The demographic data were evaluated descriptively (mean values and range).

### Ethics approval

All methods were performed in accordance with the relevant guidelines and regulations. The study received ethical clearance from the Ethics Committee of the German Society for Nursing Science in December 2019 (No. 19 − 018). All participants signed an informed consent.

## Results

### Description of the sample

A total of 12 participants were interviewed (Table 1).

**Table 1:** Participant´s characteristics (n = 12)

characteristics	number
**women/men**	5/7
**age**	∅ 55 (range 41-64)
**work experience**	∅ 24 years (range 10-45)
**profession**	
family doctor	1
palliative doctor	2
palliative nurse, ambulatory care	2
coordinator hospice service	2
social planner community	1
gerontologist	1
health care management assistant	1
social insurance clerk	1
speaker of a welfare organisation	1

### Categories

The analysis of the interviews led to four main categories that addressed *the formal care arrangement, the role of relatives in the care arrangement, the specifics of dementia, and restrictions on access to palliative care.*

Quotations from the participants were translated word for word from German to English by the first author and edited by a native speaker.

#### The formal care arrangement

Most of the interviewed experts were part of the formal care arrangement, which means that they provide care as professionals or paraprofessionals. This category pertains to the *principles* that guide their work, how that work is *organised* and what *limits* they see in their work.

##### Guiding principles of the professionals

The palliative care experts emphasised the special attitude they associate with their work. This attitude is characterised by the greatest possible respect for the needs, values and freedom of choice of people living with dementia and their relatives, by acknowledging the finite nature of life, addressing the end of life, as well as reflecting on their own limitations.

With regard to people living with dementia, experts formulated their tasks and goals to avoid overcare, control symptoms and promote well-being. When considering the end of life, experts particularly believed that measures and therapies should be critically questioned and reviewed for their contribution to achieving the treatment goal, which should be jointly determined by all those involved. The task of palliative care providers is then *“to ask the right questions*. (…) *What will happen? Will it mean going into hospital? What would it mean now if the person has a PEG inserted? [author´s note: PEG = percutaneous endoscopic gastrostomy] Is that really so necessary?* (D8A37233, 38). Symptom control, e.g., the treatment of distressing conditions such as pain, nausea or shortness of breath, which can often occur at the end of life, is generally a central concern of palliative care. Symptoms affecting people living with dementia that were frequently mentioned by the interviewed nurses and physicians were pain and agitation. In addition to the treatment of these symptoms, the promotion of well-being through biography- and needs-oriented interventions with the help of music, touch or empathic presence was of particular importance.

Another principle mentioned by the participants was that of family-centeredness. Experts support relatives through counselling and accompaniment. Counselling topics in the last phase of life mainly include the possibilities of relief, for example through care services or the use of volunteers. The topic of the end of life of the person living with dementia is rarely the subject of counselling talks, because, from the perspective of some participants, these conversations require courage on the part of the professionals to actively seek out the conversation, based on what they sense is the right time. These skills are attributed to palliative care professionals in particular.

Finally, most participants agreed that caring for people living with dementia at the end of their lives constitutes a joint task that involves different actors, including their relatives and therefore follows the principles of multi-professionalism. General practitioners (GP) play a key role in the care arrangement because they usually are the ones who know the patients best and must be prepared to involve other actors in the care by prescribing palliative care. Clear responsibilities and agreements and mutual acceptance are prerequisites for good cooperation.

##### Organisation of the formal care arrangement

Palliative care is a specialised form of care that all seriously ill and dying people are eligible to receive: *“Well, every individual has a right to palliative care. It’s a legal entitlement.“* (C4BBF662, 54). The experts emphasised that palliative care is only provided in addition to primary care, for example by the GP or a home care service. Palliative care is characterised by sufficient time resources, constant accessibility and staff continuity. The experts listed a variety of structural resources available for outpatient palliative care in Germany. These include general practitioners and palliative doctors, palliative networks, outpatient hospice services, counselling centres, and outpatient nursing services with a focus on palliative care. Additionally, requirements are made on the qualifications in the field of palliative care of those caring for patients, but these are not always fulfilled. Additional expertise on dementia is hardly available, as reported by the participants.

So that these services be of real benefit to people living with dementia, it is necessary that the need for palliative care be fully recognised and valued.  The additional palliative care services are only financed by health insurance providers if there is a clear medical indication for the prescription of palliative care by the GP, e.g., the life expectancy of the person living with dementia is only a few days, weeks or months, or the person is in an advanced stage of dementia.

In some regions of Germany, patient care is provided through a palliative network that offers psychosocial support for people living with dementia and their relatives in addition to medical and nursing care. However, relatives often feel that the services paid for by their health insurance are insufficient, and they organise additional care services at their own expense, e.g., 24-hour staff or night care, as an expert reports: “*What is not financed are really the night-care services, which I just mentioned. The families have to pay for those themselves”* (C4BBF662, 160).

##### Limits of professional support

Some experts argued that it should be carefully considered whether palliative care is necessary or whether support from a broad network of help is sufficient. They did not assume “*that all people need specialised dying-care (…), but (…) that dying, supported by voluntary workers and neighbourly help and civic solidarity and support for the person’s environment also has a value of its own, which cannot be represented in service units or the like that can be billed to the health insurance fund”* (EF399CBF, 106).

Occasionally, false expectations placed on palliative care have also led to it being seen as responsible for tasks that are not part of what it usually accomplishes; these expectations also develop because it is better funded. Furthermore, the expectation that palliative care makes dying peaceful and free of suffering cannot always be fulfilled. Finally, the services provided by the formal care system for palliative treatment and accompaniment end with the death of the person who had dementia, which also means that all support ends for the relatives, who have played a central role in the care until then. This situation occurs because care services, hospice services or GPs participate in one final conversation at most. For caring relatives, however, the death of the person living with dementia does not mean that their lives are suddenly unburdened and meaningful again. Especially if the care of the person living with dementia has taken place over a long period of time and was experienced as meaningful and rewarding for the family members, participants expressed that they still needed support (e.g., through bereavement groups) to adjust to the new situation and to cope with their grief.

#### The role of relatives in care arrangements

According to the experts, it is not possible to care for people living with dementia at home until the end of their lives without the support of the family. Experts believe that relatives generally hold a fixed position in the care of a person living with dementia, especially if this care takes place in their own home. Traditionally, palliative care essentially includes supporting and working with relatives. For people living with dementia, who are often no longer able to speak for themselves and express their wishes and needs due to their illness, relatives are an important *resource* for the person living with dementia but also for the formal carers. Especially at the end of life, decisions about invasive measures can become important, and people living with dementia can no longer make these decisions for themselves. Thus, the relatives, as the *responsible persons*, have the task of making decisions on behalf of the patient, for example about treatment. The experts also witness the enormous burden relatives face when caring for a person living with dementia, especially at the end of his or her life. Therefore, the caring relatives are also always co-respondents and the offer to resort to professional carers is also directed more or less towards the relatives to relieve them.

##### Relatives as a resource

Professional support and treatment are mainly focused on the person living with dementia. However, due to their impairments, persons with dementia are often no longer able to communicate their complaints or needs in a way that is understandable to those treating them. Thus, relatives are often the first point of contact for those treating the person living with dementia in regard to his or her care. Especially at the end of life, symptoms such as agitation or pain can occur that negatively affect the quality of life of the person living with dementia. Relatives who have known the person living with dementia for a long time can notice even the smallest changes in their behaviour or facial expressions: *“the relatives know that if that is the case, then he is in pain”* (A3BFA861, pos. 22). In relation to the assessment of the condition and needs of the person living with dementia, the family carers are *“of course the experts, also for their relatives, definitely”* (365DA0DD, 65). Relatives are also often directly involved in care by being present around the clock or by taking over nursing and care tasks. From the experts’ perspective, relatives are also the ones who take the lead in organising care at home. Family carers thus take on a key role in the care of the person living with dementia because “*without their help nothing would work, without their help this person could not stay at home*” (E63C6903, 78).

All interviews revealed that relatives were clearly emphasised as resources. Especially in the last phase of life of the person living with dementia it seems to depend primarily on the resilience and commitment of their relatives as to whether it is possible to care for that person at home until the end. The experts working in hospice care, therefore, understood that their task was predominantly to provide relief to the relatives.

##### Relatives as responsible persons

Families often want to enable their loved ones to die at home. However, the last phase of life can bring entirely new challenges for relatives due to new, mostly somatic limitations, which can lead to changes in the person living with dementia that trigger the necessity for a variety of decisions for which the relatives are not always well prepared. These decisions include, for example, decisions about artificial feeding if the person living with dementia is limited in his or her eating capacity or no longer wants to eat. Likewise, complaints may arise in the last months or weeks of life that raise the question of whether, for example, hospital admission or invasive measures are sensible and reasonable. Frequently, relatives are also legal guardians. However, even when they do not have formal power of attorney, they are involved in decisions because of their position in the care arrangement because “*it would be a bad decision not to include the wife, for example, even if she does not have power of attorney*” (365DA0DD, 131). Even if people living with dementia receive good palliative care, relatives are solely responsible for the care of the person living with dementia for many hours of the day. Despite prior agreements with the palliative care team about what should happen when the dying phase begins, in the view of the experts, the overburdening of relatives prevails in crisis situations. One expert describes the situation of the relatives as follows: “*I, as a relative or responsible person, am often alone at home with the person, and I have to endure the situation when there is shortness of breath, […] when there are the first signs of dying perhaps. As a relative, I am partly responsible*” (365DA0DD, 111). In these situations, the relatives are often afraid of doing something wrong and “*that, in this situation, they decide whether the person dies or not*” (D8A37233, 163). The experts experience that responsibility as a burden that the relatives must bear and see their task as supporting and relieving the relatives in difficult decisions.

##### Relatives as affected persons

The experts see the relatives not only as a resource but also as affected persons. For some of these experts, this realisation also means aligning part of their professional care with the needs of the relatives by informing and guiding them or providing them with emotional support.

Family carers are described by the experts as being very stressed. Burdens arise when the caring relatives are old themselves and when direct care is provided, which is also a physically demanding situation, especially in the last phase of life. Then, the relatives “*do quite a lot for their age, because they are often over 80 […] And for that matter they certainly have health problems themselves under certain circumstances. Yes. That is, well, in the care it is difficult, yes”* (A3BFA861, 252–254). To ensure proper care around the clock, relatives often have to use their own financial resources, for example, to organise care at night. For younger carers, other social roles, such as being fathers/mothers or employees, also contribute to the burden. The uncertain duration of care constitutes another burden. The family members’ resilience often ends when the relative with dementia shows so-called challenging behaviours: “*some relatives then simply can’t take it anymore*” (A3BFA861, 188).

For some experts, relatives need the attention and support of the care system not only during the entire time they are carers but also, and in particular, in the last phase of the life of the person living with dementia because that is when they must face diverse tasks and burdens. Even after the death of the person living with dementia, which for relatives means the end of an often long-term caring relationship, relatives need support as they grieve. Relatives sometimes find it difficult to accept the end of life, and “*this acceptance of the changing state (…) is sometimes accompanied by a loss of meaning—so, for relatives, this is of course accompanied by the fact that they have to adapt again; all these things have to be accommodated for. And I don’t know who will do that. Who is there?”* (D8A37233, 72).

#### The specifics of dementia

The principles and organisation of the formal care arrangement and the role of relatives represent general framework conditions of palliative care. These conditions are not exclusively characteristic of the situation of people living with dementia at the end of life. The category “specifics of dementia”, which is presented below, describes the specific challenges that arise, particularly in the care and support of people living with dementia at the end of life. The experts see these challenges first in the *limited communication* with people living with dementia. *The gradual and long course of the disease* and the *unclear characteristics* of the last phase of life also render timely palliative care difficult. These difficulties also lead to *ethical challenges* that confront all those involved.

##### Limited communication

Especially in the last phase of life, discomfort or pain can occur. If people living with dementia cannot communicate about these ailments themselves, special attention and observation by relatives or formal carers are needed. The experts mention that recognising pain in people living with dementia is particularly challenging. Proxy assessment instruments for the detection of pain are either not known or cannot be timely implemented because they are too comprehensive. One expert describes the problem as follows: *“And um, there are other ways of recognising pain, but these are often so time-consuming for nursing staff, so if you really have to work through a list like this, how are the facial expressions, how are the gestures, how do the facial expressions change when turning, when positioning the patient; it is so time-consuming that I often have the feeling, no, I really know, that the nurses hardly work with it at all, because they simply cannot incorporate it into their time frame”* (C4BBF662, 64).

For people living with dementia it is also difficult to communicate their wishes, needs or fears as the disease progresses. Especially with regard to decisions that may have to be made at the end of life, the increasing impairment of communication represents a major obstacle. Relatives and professional carers are then dependent on the determination of the presumed will of the person living with dementia. Living wills or early discussions about end-of-life wishes at an early stage of dementia would facilitate this task. However, directives are not always available, and people living with dementia often miss the right time for a conscious discussion about their own finiteness.

##### Gradual course of the disease

The experts describe dementia as protracted and gradual, especially in comparison with tumour-induced diseases. The state of health of cancer patients can deteriorate rapidly and require palliative care as crisis intervention. In people living with dementia, however, the illness develops over the long-term and leads to a *“medium-length dying over a longer period of time”* (EF399CBF, 58). These long, sometimes fluctuating courses of the illness make it difficult to recognise the last phase of life or to start palliative treatment in time. This situation can lead to people living with dementia being accompanied for years, for example by volunteers of outpatient hospice services; it also leads to the need for palliative care not being recognised due to the absence of a crisis situation. For one interviewed expert, the diagnosis of dementia alone is not a sufficient justification for palliative care: *“Dementia alone would not be enough for me to say that palliative care was necessary. We have the Alzheimer’s groups; we have all the institutions that have been working well for years, decades”* (D8A37233, 38).

##### Unclear characteristics of the last phase of life

Palliative care for people living with dementia, when delivered through patient- and family-oriented care, is often part of the treatment by the general practitioner and nursing care in outpatient care services. For more specialised palliative care involving palliative experts, it is necessary that professionals or relatives perceive that the person living with dementia is approaching the end of life and that care may need to be adapted to this situation. Experts agree that the characteristics of the last phase of life for people living with dementia are not clearly defined and may not be defined. Some recommend an orientation towards the severity of dementia; others consider that “classic” palliative symptoms such as physical deterioration and increasing weakness and somatic complaints, as well as confusion and agitation, are characteristic. Often, however, the intuitive abilities of the carers are needed to assess the phase of life of the person living with dementia.

From the perspective of health insurance, the clear predictability of the end of life is a prerequisite for the financing of palliative care. However, the prediction of a very limited life expectancy of a few days or weeks does not seem to fit the situation of people living with dementia. One expert suggests the necessity to “*perceive palliative in a different way and really not define this time period so narrowly, but to look at a different temporal form of life span”* (EF399CBF, 40).

##### Ethical challenges

One of the challenges that is frequently raised in the interviews is the question of nutrition at the end of life. From the perspective of palliative care providers, giving fluids and food at the end of life prolongs the dying process and should therefore be avoided. Relatives express their fear of being responsible for the death of their loved one by asking: “*am I letting my relative starve to death?*” (A3BFA861, 46). Since the dying person often cannot be asked about their own wishes due to their dementia, tensions can sometimes arise based on the conflicting priorities of relatives and palliative care providers. In these cases, ethical discussions are also conducted to determine the presumed will of the person living with dementia.

Under certain circumstances, a living will facilitates treatment according to the wishes of the person living with dementia. However, living wills are often either not available or, in the view of the experts, not very meaningful. This lack of meaningfulness emerges because there is often a lack of professional support and advice, for example, from the GP, who could explain the consequences of the decisions made. Moreover, some experts observe that even people in the advanced stages of dementia can feel happiness and contentment despite a condition that would have seemed unbearable to them at the onset of their dementia. Thus, the wishes established in the past may change in the course of the dementia but those wishes can no longer be communicated.

#### Restrictions on access to palliative care

Although all people with a life-limiting illness have a right to palliative care, there are nevertheless mainly structural barriers for people living with dementia that can hinder adequate end-of-life care. Palliative care experts often encounter *closed systems* of (primary care) care for people living with dementia that hardly let them enter. The *lack of awareness* among caregivers about the potential of palliative care for people living with dementia also prevents timely palliative care at the end of life. Finally, the participants also complain about *limited resources* for palliative care in the outpatient setting.

##### Closed systems

Owing to their age and accompanying illnesses, people living with dementia require a network of informal and formal carers for several years. This care network usually includes the GPs, who are the first points of contact for medical issues and who have often known and treated their patients for many years. The outpatient care service is also part of this care network. Careful observation of the condition of the person living with dementia by professional caregivers and their willingness to change established routines are required to recognise the palliative needs of patients and adapt the care accordingly.

When the need for palliative treatment and support is identified in a person living with dementia, additional palliative care experts must expand the existing care network. However, palliative physicians in particular experience the long-established care provided by the general practitioner as closed care, which additional care providers perceive as a threat or competition; moreover, *“(…) where there is competition, walls are erected, that is it somehow—yes”* (EF399CBF, 46). It seems that GPs in particular are worried that they will be pushed out of palliative care altogether. In addition to disputes about competence, financial reasons obviously also play a role: “*it’s a bit like taking away the sandwich “(*A7D23AEC, 101). However, adequate, cooperative care at the end of life can only succeed if the palliative experts also respect the long-standing, trusting relationships between GPs/nursing services and people living with dementia and see themselves as supporting and complementing the existing care because the *“palliative is actually an add-on”* (D8A37233, 56). Sometimes, therefore, the unwillingness to adapt work processes or to cooperate collegially with experts in palliative care prevents the palliative care of a person living with dementia at the end of life from taking place.

##### Lack of awareness of palliative care among care providers

Although participants consider palliative care for people living with dementia to be useful and effective, only a few of the palliative care patients in outpatient care have dementia—*“at most 10%”* (70,844,840, 48). Similarly, people living with dementia in the last phase of life are rarely treated in a palliative care unit or hospice. If care at home is no longer possible, they are transferred to a nursing home. The reasons for not considering the palliative needs of people living with dementia include that the palliative structures in Germany are very strongly oriented towards cancer patients. This situation also means that palliative care providers are not dementia experts and thus lack knowledge, for example, about how to recognise pain in people living with dementia, or about how to predict a limited life expectancy or make specific psychosocial offers. The low proportion of people living with dementia among outpatient palliative care patients is also explained by the fact that palliative care is not the focus of outpatient care. In outpatient medical care for people living with dementia, the focus is mainly on diagnosis and initial measures. The dying phase is only rarely addressed.

Experts believe that the uncertainty and fear of caregivers in verbalising the topic of end of life also prevents timely planning of care for the dying phase. Moreover, outpatient (nursing) care lacks concepts to deal with people living with dementia at the end of their lives: *“So there is still uncertainty, a certain inability to communicate. So, I think there is still a lack of concepts”* (E189831F, 34).

##### Limited resources

Although there are many resources available for outpatient palliative care, the experts also report overburdened outpatient structures, staff shortages, lack of coordination and regional gaps in care, especially in rural areas. Against the background of limited resources, people living with dementia seem to be more likely to fall out of the focus of palliative care than, for example, people with cancer. The sometimes insufficient funding of outpatient palliative care also represents a possible access barrier for people living with dementia. If necessary services provided by professionals are not all financed, care depends on the commitment of individuals. Only a few years ago, it was still apparently common practice for health insurance companies to generally refuse to cover the costs of palliative care for non-oncological patients. However, this practice has changed in recent years for the better for people living with dementia. Finally, a limiting aspect is also the insufficient relief of the relatives. *“The fact that people with dementia often do not die at home (…) is certainly largely due to the fact that the relatives reach the limits of their resilience”* (365DA0DD, 99). Experts consider that the relief offered to caring relatives, such as care services, is underfunded. Especially at the end of life, 24-hour care is necessary, which relatives can only finance by spending private funds.

## Discussion

The results of the study have provided insight into the organisation of end-of-life care for people living with dementia in Germany and the role that relatives play in end-of-life care. In particular, the participants made clear that they experienced barriers to end-of-life care due to the specifics of dementia and other factors.

Palliative care for people living with dementia.

People living with dementia, like all people with a life-limiting illness, have a right to palliative care. This type of care is characterised by patient- and family-centeredness and multi-professionalism. To meet the needs of dying people for pain relief and further discomfort relief, psychological and spiritual support, social participation, and self-determination, the support of a team consisting of experts and volunteers is necessary. The support of this team includes the needs and support of the patient’s family [32]. Palliative care, which is an established routine for patients with cancer, for example, and for which structures and resources are available in Germany, faces specific challenges in the care of people living with dementia. Since people living with dementia are no longer able to communicate their wishes and needs, those treating them must have the skills and measures to perceive their needs. In our study, the experts reported that these skills are not always present. Studies on barriers to palliative care also point to a lack of skills in managing symptoms, such as pain, or psychological/social aspects, as well as difficulties in recognising and treating anxiety and depression [33, 34].

Experts have different opinions about when people living with dementia should receive palliative care [35, 36]. One recommendation derived from these findings is that the initiation of palliative care should not be dependent on the stage of the illness or cognitive and functional decline but should allow flexibility, so that palliative care is based primarily on the needs of people living with dementia [35, 37]. This approach is in line with a person-centred approach that has long been recommended for the care of people living with dementia. To initiate end-of-life care, it is necessary to recognise when the dying phase begins. This assessment is often not easy in people living with dementia, as the interviews showed. International studies have also come to this conclusion, indicating that there is no common definition for the last phase of life and that different instruments are used to record it [35, 36].

Ethical challenges often arise regarding the necessity or appropriateness of artificial nutrition for people living with dementia in the last phase of life. Guidelines recommend that the decision to use artificial feeding should be made on an individual basis, balancing expected benefits with potential burdens, prognosis and patient will [38].

### The importance of relatives

From the perspective of the interviewed experts, the care of people living with dementia at the end of life in their home is not possible without the willingness and participation of relatives. Relatives are those who spend the most time with the person living with dementia, coordinate care, make decisions and usually know the person living with dementia best. For the experts, relatives are also often the first point of contact in regard to the care of the person living with dementia. However, the experts also see that the relatives who provide care face an enormous burden. The burden on relatives of people living with dementia, which can be evident on several levels—psychological, physical, financial, and social—and has been the subject of numerous studies [39, 40]. The experiences of relatives providing end-of-life care have been rarely studied. The few studies on the topic have concluded that relatives need more support in the palliative care phase but also beyond the death of the person living with dementia [17, 37, 41, 42]. The interviewed experts also addressed the insufficiency of the support relatives receive; for example, the structural or financial offers are insufficient and also the lack of openness and planning to prepare for the last phase of life. The lack of support for relatives after the death of the person living with dementia has been criticised by the experts who have indicated potential improvements in palliative care. Studies could show that when a person living with dementia dies, caring relatives face a difficult time of grief and bereavement, during which adjustments are necessary to adapt to changing roles, changing time management, new routines and a return to a social life [17, 43]. Relatives need support for these tasks [41].

Finally, the participants considered a broader social support network as necessary to protect relatives from being overwhelmed, especially in the last phase of the patient’s life. This support network can also help in overcoming isolation and loneliness, even beyond the death of the person living with dementia [37] and represents a decisive factor that makes it possible for people living with dementia to die at home [18].

### Difficult access to palliative care

In terms of access to palliative care, in Germany, GPs play a central role. They usually know the patients with dementia, their medical history and their families well. Moreover, they are the ones who must recognise the need for palliative care and treatment and be willing to involve other actors in the patient’s care. However, the results of this study indicate that GPs pay little attention to the special importance and challenge of caring for people living with dementia in the last phase of life. The low level of attention to that last phase of life is also reflected in the fact that this phase is often not addressed early enough and the wishes of the person living with dementia are rarely documented through an advance directive, for example. The GP could have an essential role here by advising the person living with dementia and supporting him or her in establishing an advance directive. A recent review concluded that a lack of confidence and skills, in particular, is a barrier to palliative care by GPs [33].

Likewise, the medical care provided by a GP seems to be inadequately designed for interdisciplinary cooperation. International studies have also found that there are barriers to palliative care for people living with dementia. These include, for example, lack of cooperation in the health system [44–46], attitudes, beliefs and values of health professionals [46, 43], lack of knowledge [44] or lack of understanding that dementia is an incurable disease and a palliative condition [47, 48].

## Limitations

The results of this study are not transferable to the overall care situation of people living with dementia at the end of life in Germany, as they are based on the experiences of only 12 experts. Participants were recruited through gatekeepers from the researcher’s professional network, which may have led to bias. However, they provide valuable indications of possible challenges and potential for improvement and are in line with results found in international studies. Further data collection is necessary, especially from people living with dementia and their relatives, to record the care situation in Germany. Furthermore, because the first author solely performed the analysis of the data, the validity and credibility of that analysis may have been impacted.

## Conclusions

Overall, the study showed that, from the perspective of professionals, palliative care could make a positive contribution to the care of people living with dementia in the last phase of their lives. However, a person-centred approach, which bases the initiation of palliative care on the needs of the person living with dementia rather than on the stage of the disease, seems to make sense. Although general palliative care in Germany is well organised, there is a need for improvement in end-of-life care for people living with dementia. In particular, specific care concepts and resources are needed to overcome barriers so that people living with dementia can also benefit from good palliative care, and their relatives can be supported in the best possible way.

### Electronic supplementary material

Below is the link to the electronic supplementary material.

Supplementary Material 1

## Data Availability

Owing to confidentiality agreements with research participants, supporting data can be made available for some but not all of the interviews via reasonable request to the corresponding author.
