# Supplementary material for: Home-based care for people living with dementia at the end of life: the perspective of experts
Source: BMC Palliat Care. 2023 Sep 1;22:123. doi: 10.1186/s12904-023-01251-z (PMC10472677; doi:10.1186/s12904-023-01251-z)
Supplement: Supplementary file 1 — Supplementary Material 1 [file 12904_2023_1251_MOESM1_ESM.docx]

| **Questions** | **Probes** |
| --- | --- |
|  |  |
| **Introduction** |  |
| What are your experiences with end-of-life care for people with dementia? | To what extent are you in touch with the topic/the care of people with dementia in your work? |
|  |  |
| **Access to palliative care** |  |
| According to international literature, far fewer people with dementia receive palliative care than people with cancer, for example. Is this also true for Germany? | Why do you think that is? |
|  |  |
| How do you assess access to palliative care for people with dementia? |  |
|  |  |
| Is palliative care for people with dementia at all meaningful and effective? |  |
|  |  |
| **Characteristics of the last phase of life** |  |
| How can you recognise the last phase of life in a person with dementia? |  |
|  |  |
| What is characteristic of the last phase of life for people with dementia? |  |
|  |  |
| **Challenges in the last phase of life** |  |
| Do you think palliative care is necessary for people with dementia in the last phase of life? | Why?  Why not? |
|  |  |
| What challenges arise in the care of people with dementia in the last phase of life? | Could you please give an example of this?  Are there differences in palliative care for cancer patients, for example? If so, what are they? |
|  |  |
| What challenges arise when the person with dementia wants to live at home until the end? | What challenges does this pose for the palliative care team? |
|  |  |
| Do professionals and volunteers need special knowledge and skills when caring for people with dementia at the end of life? | Where can you get them? |
|  |  |
| **Use of palliative care services** |  |
| Do people with dementia use palliative care services in the same way as people with other life-limiting illnesses? | Why not? |
|  |  |
| **Wishes of relatives and people with dementia in the last phase of life** |  |
| What are the needs and wishes of relatives and people with dementia in the last phase of life? | Are these needs different from the needs of relatives and people affected by other diseases? |
|  |  |
| **Barriers and facilitators for remaining at home** |  |
| Is it possible to care for people with dementia in the last phase of their lives in their own homes? |  |
|  |  |
| What conditions does end-of-life care at home require? | Are these conditions always met? |
|  |  |
| **Role of relatives** |  |
| What is the role of family members of people with dementia in end-of-life care? |  |
|  |  |
| **Decision-making** |  |
| What role do relatives and people with dementia play in end-of-life decision-making? |  |
|  |  |
| **Support for relatives in the last phase of life** |  |
| What support do relatives need in the last phase of life? |  |
|  |  |
| **Final question** |  |
| In a perfect world, what would palliative care in the home for people with dementia look like? |  |
